# Healthy aging among community-dwelling older adults aged ≥60 years in China: results from a 2023 cross-sectional study

**DOI:** 10.3389/fpubh.2026.1749930

**Published:** 2026-04-10

**Authors:** Hui Li, Li Gao, Yan Gao, Hanxue Zhang, Shuxian Yang, Liang Chang, Minjie Qi, Rui Wen, Yi Wang, Lei Fan, Linqi Diao

**Affiliations:** 1 Institute for Prevention and Control of Chronic Non-communicable Diseases, Henan Provincial Center for Disease Control and Prevention, Zhengzhou, Henan, China; 2 Kaifeng Center for Disease Control and Prevention, Kaifeng, Henan, China; 3 Pingdingshan Center for Disease Control and Prevention, Pingdingshan, Henan, China

**Keywords:** China, correlates, cross-sectional study, healthy aging, older adults

## Abstract

**Background and aims:**

With the global population aging, this study aimed to estimate the prevalence of healthy aging and its associated factors in China.

**Methods:**

This cross-sectional study was conducted in Henan Province, China, in 2023. A multi-stage, stratified, probability-proportional-to-size (PPS) sampling method was employed to enroll 9,180 community-dwelling older adults aged ≥60 years. Healthy aging was defined as “no major chronic diseases,” “no disability,” “high cognitive function,” “high physical function,” and “active social engagement.” The weighted prevalence of healthy aging was evaluated. Weighted Poisson regression models were used to estimate the prevalence ratios (*PR*s) for the association between sociodemographic/lifestyle factors and healthy aging.

**Results:**

The overall weighted prevalence of healthy aging was 6.1%. The weighted prevalence of no major chronic diseases, no disability, high cognitive function, high physical function, and active social engagement was 23.7, 82.2, 42.4, 39.0, and 71.8%, respectively. The *PR* of healthy aging was inversely associated with age (*PR* = 0.65 in those aged 70–79 years and *PR* = 0.30 in those aged ≥80 years). Older adults residing in urban areas had a higher *PR* of healthy aging than those in rural areas (*PR* = 1.44, 95% *CI* 1.30–1.59). Higher education, higher income, alcohol consumption, and physical activity were all positively associated with healthy aging (*p* < 0.05).

**Conclusion:**

The prevalence of healthy aging among community-dwelling older adults in Henan Province was low. Fewer than half of the participants were free from major diseases or maintained high cognitive and physical function. Promoting healthy aging requires a multifaceted approach that integrates healthcare, economic, and educational initiatives. Particular attention should be given to female individuals, rural residents, individuals with lower education and income levels, and individuals who are divorced, widowed, cohabiting, or living alone.

## Introduction

China is undergoing one of the fastest demographic transitions toward an aging society globally. The proportion of adults aged ≥60 years increased from 13.3% in 2010 to 18.7% in 2020 (1, 2). This unprecedented demographic shift poses substantial challenges to public health and social welfare systems, particularly in economically underdeveloped regions. Henan Province, a traditional agricultural region in central China, has long maintained a large population due to historical patterns of high fertility rates. The Seventh National Population Census reported 99.36 million residents, of whom 17.96 million (18.1%) were aged ≥60 years (2). Notably, the proportion of older adults residing in rural areas of Henan is substantially higher than the national average (44.6% vs. 36.1%) (3). Given that urban–rural residence is a well-established social determinant of disparities in longevity, healthcare access, and healthy aging trajectories, estimating the prevalence of healthy aging in this specific context is not only critical for local policy-making but also for providing valuable insights into the challenges of healthy aging across rural China.

Therefore, promoting health in later life has become a pressing societal priority. The World Health Organization (WHO) defines healthy aging as “the process of developing and maintaining functional abilities that enable well-being in older age,” including the ability to meet basic needs, learn, grow, make decisions, maintain mobility, build and nurture relationships, and contribute to society (4–6). To advance this goal, the WHO proposed a global strategy and action plan for healthy aging (2016–2020), involving governmental and non-governmental participants, age-friendly environments (spanning society, community, and family), health and long-term care systems, and standardized approaches for monitoring and evaluating healthy aging. Subsequently, 10 priorities were established to guide the UN Decade of Healthy Ageing (2021–2030), with particular emphasis on translating healthy aging research into targeted policies, especially in developing countries and regions (7). Despite this global policy framework, the operational definition of healthy aging remains inconsistent in epidemiological research. Two predominant frameworks coexist: The contemporary WHO model, which emphasizes functional ability within environmental contexts (4), and the earlier Rowe and Kahn model, which defines healthy aging as the simultaneous avoidance of major diseases, maintenance of high cognitive and physical function, and active social engagement. The Rowe and Kahn model has been extensively applied in large-scale population studies due to its operational clarity, revealing substantial cross-national variation in prevalence. For instance, the prevalence rate is 10.9% in the US (8), 8.5% across European countries (9), 25.4% in Singapore (10), 17.6% in Japan, and as high as 62.0% in Brazil (11). Within China, prevalence estimates have also varied considerably, depending on age thresholds (≥60 vs. ≥65 years) and sample sources (community-dwelling vs. institutionalized older adults) (12, 13). However, province-level estimates remain scarce, particularly in regions with large rural populations. To address this gap and generate policy-relevant evidence for aging populations in rural-predominant settings, this study aimed to evaluate the prevalence of healthy aging and its associated factors among a provincially representative sample of adults aged ≥60 years in Henan Province, China.

## Methods

### Study design and participants

Data were obtained from a cross-sectional study conducted in Henan Province, China, in 2023. The study aimed to estimate the prevalence of healthy aging and the epidemiological status of major chronic diseases, lifestyle factors, physical function, cognitive function, depression, and activities of daily living (ADLs), as well as associated factors among older adults aged ≥60 years. Data collection included face-to-face questionnaires, physical examination, and biochemical tests. The sample size was calculated using the following formula:


$$N=\text{deff}\frac{u_{a}^{2}p(1-p)}{d^{2}}$$

The parameters were defined as follows: a two-sided significance level (*α*) of 0.05 (*u* = 1.96), an estimated prevalence of healthy aging (*p*) of 15.6% based on a previous study (13), a relative error (*r*) of 20% (corresponding to an absolute error *d* = 20%*15.6% = 3.12%), and a design effect (*deff*) of 4 to account for the multi-stage complex sampling design. The initial calculation yielded a minimum required sample size of 2,079 participants per stratum. Considering stratification by urbanization level (4 strata), a potential non-response rate of 10%, and other practical considerations, a total of 9,180 participants were finally enrolled across 17 counties/districts in the whole province, with 540 participants allocated to each county/district.

A multistage, stratified, probability-proportional-to-size (PPS) sampling method was used to recruit a representative sample of older adults aged ≥60 years who had resided in the area for at least 6 months in the past year. Participants were excluded if they (i) were aged <60 years; (ii) resided in barracks, dormitories, or nursing homes; (iii) had severe mental disorders; (iv) had communication barriers (e.g., deafness or hearing impairment) that prevented them from completing the questionnaire; or (v) had high-level paraplegia that precluded the completion of biomedical measurements for physical performance and body composition. First, 17 districts/counties were selected based on the urbanization rate (3 districts and 6 counties with higher rates; 3 districts and 5 counties with lower rates). Within each selected district/county, three streets/towns were sampled using PPS sampling, with the population aged ≥60 years as the measure of size. Subsequently, two villages/communities were similarly selected from each street/town via PPS sampling. This was also based on the older population size. Finally, within each selected village/community, a total of 90 participants aged ≥60 years were recruited using simple random sampling. A total of 8,484 participants were included in the final analysis, with a response rate of 92.4%. The study was conducted in accordance with the Declaration of Helsinki, and the protocol was approved by the Ethics Committee of Henan Provincial Center for Disease Control and Prevention (Approval No. 2023-KY-006-02). All participants provided their informed consent.

### Definition of healthy aging

The conceptualization of healthy aging proposed by Rowe and Kahn (14) was used in this study. It is a multidimensional concept including five aspects, namely no major diseases, no disability, high cognitive function, high physical function, and active engagement with life. Participants who met the criteria for all five aspects were classified as experiencing healthy aging. The definitions and criteria for each indicator were based on previous studies (13, 15) and are presented below

(1) No major diseases were defined as the absence of major chronic diseases and depression. Major chronic disease was assessed by trained staff who asked participants via electronic equipment: “Have you been diagnosed with cancer, diabetes, chronic lung disease, heart disease, or stroke?” Depression was defined as a score of <5 on the 15-item Geriatric Depression Scale (GDS-15).
(2) No disability was defined as self-reported absence of limitations in any ADLs, including bathing, dressing, toileting, indoor transferring, continence, and feeding.
(3) Cognitive function was assessed using the Chinese version of the mini–mental state examination (MMSE) questionnaire, with a total score of 30. A score of ≥26 was considered indicative of high cognitive function.
(4) High physical function was defined as either self-reported participation in moderate- to high-intensity physical activities on a weekly basis or high performance on physical function tests, including the 4-meter gait speed test and the five-times-sit-to-stand test (FTSST), with completion times ranked in the highest tertile.
(5) Active social engagement was defined as self-reported participation in social activities with friends or family, engaging in outdoor physical exercise, or playing poker with friends during the past year.

### Other variables

(1) County-level variables: Residence areas were divided into urban and rural areas based on the definition provided the National Bureau of Statistics. (2) Sociodemographic variables: Education level was categorized as illiterate, primary school, junior high school, and above. Economic status was classified into three categories—low, medium, and high—based on the quartiles of income in the past year. Marital status was dichotomized into two groups: married and other statuses (never married, widowed, divorced, separated, or cohabiting). Living status was categorized as yes or no based on the question, “Do you usually live alone?” (3) Lifestyle variables: Current smoking was defined as smoking daily or occasionally at the time of the survey. Drinking was defined as alcohol consumption during the past 12 months. Dietary intake was calculated using a food frequency questionnaire (FFQ). Insufficient fruit and vegetable intake was defined as <400 g/day (16). Excessive red meat intake (including pork, beef, and mutton) was defined as >100 g/day (17). Physical inactivity was defined as spending <150 min per week on work-related (including vigorous and moderate activities) and leisure-time physical activities (18).

### Statistical analysis

SAS version 9.4 (SAS Institute, Inc., Cary, NC, USA) was used for analysis. Given the underlying structure of the older population, as well as the multistage sampling design, non-response rate, and post-stratification, standard sample weights were constructed based on the 2020 Henan Census Population. All analyses incorporated complex sample design weights derived from these factors. Continuous variables are summarized as mean ± standard deviation, and categorical variables are presented as n (percentage). The Rao–Scott *χ^2^* test was used for the comparison of categorical variables. Weighted Poisson regression models were used to estimate prevalence ratios (*PR*s) for healthy aging and its correlates.

## Results

### Basic demographic characteristics of participants

A total of 8,484 participants (47.7% male and 52.3% female individuals) aged ≥60 years were included and analyzed. The proportions of participants aged 60–69, 70–79, and ≥80 years were 54.3, 34.6, and 11.1%, respectively. Among the participants, 28.2% were illiterate, 31.7% had completed primary school education, and 40.1% had completed junior high school and higher education. The number of participants with low, middle, and high annual household income was 1,438 (17.0%), 3,203 (37.8%), and 1,257 (14.8%), respectively. Married individuals accounted for 80.5% of the sample. In total, 83.2% of older individuals did not live alone (Table 1).

**Table 1:** Basic characteristics of study participants.

Characteristics	*N*	%
All	8,484	100.0
Sex		
Male	4,043	47.7
Female	4,441	52.3
Age group (years)		
60–69	4,607	54.3
70–79	2,932	34.6
≥80	945	11.1
Residence area		
Urban	4,290	50.6
Rural	4,194	49.4
Educational level		
Illiteracy	2,391	28.2
Primary school	2,689	31.7
Junior high school and above	3,404	40.1
Annual household income*		
Low	1,438	17.0
Middle	3,203	37.8
High	1,257	14.8
Marital status		
Married	6,833	80.5
Other (divorced/widowed/cohabitation)	1,651	19.5
Living alone		
Yes	1,423	16.8
No	7,061	83.2

^*^Data are missing.

### Prevalence of healthy aging in different subgroups

The weighted prevalence of healthy aging was 6.1% among the population aged ≥60 years in Henan Province in 2023. The weighted prevalence of no major diseases, no disability, high cognitive function, high physical function, and active social engagement was 23.7, 82.2, 42.4, 39.0, and 71.8%, respectively. Significant differences in healthy aging across certain demographic characteristics are shown in Table 2.

**Table 2:** Prevalence of healthy aging and its five indicators among older adults aged ≥60 years.

Characteristics	Healthy aging	No major disease	No disability	High cognitive function	High physical function	Active social engagement
All	531 (6.1%)	1885 (23.7%)	6,952 (82.2%)	3,657 (42.4%)	3,509 (39.0%)	6,180 (71.8%)
Sex						
Male	329 (3.8%)	964 (11.7%)	3,418 (40.2%)	2,173 (25.8%)	1837 (20.4%)	3,041 (35.4%)
Female	202 (2.3%)	921 (12.0%)	3,534 (42.0%)	1,484 (16.7%)	1,672 (18.6%)	3,139 (36.4%)
Rao–Scott *χ^2^* test	*χ^2^* = 28.61, *p* < 0.001	*χ^2^* = 2.53, *p* = 0.112	*χ^2^* = 19.01, *p* < 0.001	*χ^2^* = 289.34, *p* < 0.001	*χ^2^* = 33.20, *p* < 0.001	*χ^2^* = 17.78, *p* < 0.001
Age group (years)						
60–69	381 (4.6%)	1,132 (14.7%)	4,003 (48.3%)	2,262 (27.3%)	2,176 (24.9%)	3,373 (39.8%)
70–79	130 (1.3%)	579 (6.6%)	2,320 (25.3%)	1,156 (12.1%)	1,132 (11.6%)	2,124 (22.7%)
≥80	20 (0.2%)	174 (2.4%)	629 (8.7%)	239 (3.0%)	201 (2.5%)	683 (9.3%)
Rao–Scott *χ^2^* test	*χ^2^* = 70.88, *p* < 0.001	*χ^2^* = 32.06, *p* < 0.001	*χ^2^* = 163.89, *p* < 0.001	*χ^2^* = 175.96, *p* < 0.001	*χ^2^* = 158.05, *p* < 0.001	*χ^2^* = 0.81, *p* = 0.666
Residence area						
Urban	361 (3.9%)	955 (10.8%)	3,585 (36.8%)	2,240 (23.3%)	2030 (19.8%)	3,305 (32.6%)
Rural	170 (2.2%)	930 (13.0%)	3,367 (45.4%)	1,417 (19.1%)	1,479 (19.2%)	2,875 (39.2%)
Rao–Scott *χ^2^* test	*χ^2^* = 57.19, *p* < 0.001	*χ^2^* = 2.96, *p* = 0.085	*χ^2^* = 21.77, *p* < 0.001	*χ^2^* = 229.47, *p* < 0.001	*χ^2^* = 82.34, *p* < 0.001	*χ^2^* = 22.71, *p* < 0.001
Educational level						
Illiteracy	17 (0.3%)	496 (6.7%)	1827 (23.5%)	219 (2.8%)	666 (8.2%)	1,627 (20.7%)
Primary school	119 (1.6%)	520 (6.8%)	2,133 (25.4%)	1,036 (13.0%)	1,006 (11.3%)	1921 (22.2%)
Junior high school and above	395 (4.2%)	869 (10.2%)	2,992 (33.3%)	2,402 (26.6%)	1837 (19.5%)	2,632 (28.9%)
Rao–Scott *χ^2^* test	*χ^2^* = 151.54, *p* < 0.001	*χ^2^* = 18.38, *p* < 0.001	*χ^2^* = 90.82, *p* < 0.001	*χ^2^* = 1454.84, *p* < 0.001	*χ^2^* = 257.90, *p* < 0.001	*χ^2^* = 41.63, *p* < 0.001
Annual household income						
Low	44 (0.5%)	269 (3.7%)	1,101 (14.7%)	403 (5.4%)	474 (6.0%)	1,034 (14.3%)
Medium	234 (3.1%)	806 (11.0%)	2,689 (34.2%)	1,533 (19.4%)	1,346 (16.4%)	2,324 (28.7%)
High	173 (1.7%)	329 (3.5%)	1,147 (11.7%)	864 (8.5%)	767 (7.4%)	1,049 (10.3%)
Rao–Scott *χ^2^* test	*χ^2^* = 123.00, *p* < 0.001	*χ^2^* = 45.04, *p* < 0.001	*χ^2^* = 99.22, *p* < 0.001	*χ^2^* = 351.18, *p* < 0.001	*χ^2^* = 168.75, *p* < 0.001	*χ^2^* = 47.36, *p* < 0.001
Marital status						
Married	462 (5.4%)	1,565 (19.9%)	5,678 (67.9%)	3,112 (37.0%)	2,925 (33.0%)	4,946 (57.7%)
Divorced/widowed/cohabitation	69 (0.7%)	320 (3.8%)	1,274 (14.3%)	545 (5.5%)	584 (6.0%)	1,234 (14.1%)
Rao–Scott *χ^2^* test	*χ^2^* = 12.69, *p* < 0.001	*χ^2^* = 7.47, *p* = 0.006	*χ^2^* = 26.98, *p* < 0.001	*χ^2^* = 99.66, *p* < 0.001	*χ^2^* = 26.10, *p* < 0.001	*χ^2^* = 7.46, *p* = 0.006
Living alone						
Yes	74 (0.9%)	273 (3.7%)	1,132 (13.6%)	503 (6.1%)	536 (6.1%)	1,071 (13.0%)
No	457 (5.2%)	1,612 (20.1%)	5,820 (68.6%)	3,154 (36.3%)	2,973 (32.9%)	5,109 (58.8%)
Rao–Scott *χ^2^* test	*χ^2^* = 0.97, *p* = 0.324	*χ^2^* = 3.97, *p* = 0.046	*χ^2^* = 8.64, *p* = 0.003	*χ^2^* = 23.34, *p* < 0.001	*χ^2^* = 6.32, *p* = 0.012	*χ^2^* = 7.52, *p* = 0.006

The prevalence of healthy aging showed a declining trend with increasing age (4.6% among those aged 60–69 years, 1.3% among those aged 70–79 years, and 0.2% among those aged ≥ 80 years). It was higher in male individuals than in female individuals (3.8% vs. 2.3%, *p* < 0.001) and was also higher in urban residents than in rural residents (3.9% vs. 2.2%, *p* < 0.001). Regarding socioeconomic factors, the prevalence was highest among older adults with junior high school education or above (4.2%, *p* < 0.001) and was also higher among married individuals (5.4%, *p* < 0.001) and those who did not live alone (5.2%, *p* < 0.001).

### Associations between healthy aging and its correlates

The *PR*s of healthy aging were inversely associated with age. Compared to participants aged 60–69 years, the *PRs* were 0.65 (*PR* = 0.65, 95% *CI* 0.58–0.73) for those aged 70–79 years and 0.30 (*PR* = 0.30, 95% *CI* 0.23–0.40) for those aged ≥ 80 years. Higher educational levels were positively associated with healthy aging. Compared to illiterate participants, the *PR*s were 4.89 (*PR* = 4.89, 95% *CI* 3.85–6.23) for those with primary school education and 7.06 (*PR* = 7.06, 95% *CI* 5.56–8.97) for those with junior high school education or above. Similarly, participants with medium and high annual household income levels had significantly higher *PR*s of healthy aging (*PR* = 1.99, 95% *CI* 1.72–2.29; *PR* = 2.20, 95% *CI* 1.88–2.57, respectively) compared to those with low income levels.

Regarding lifestyle factors, participants who reported current alcohol consumption and sufficient physical activity had higher *PR*s for healthy aging, while insufficient intake of fruits and vegetables negatively impacted healthy aging. No significant associations were observed between sex, marital status, current smoking, excessive red meat intake, and healthy aging (Table 3).

**Table 3:** Associations between healthy aging and its correlates.

Variables	Model 1 (*PR* 95% *CI*)	Model 2 (*PR* 95% *CI*)	Model 3 (*PR* 95% *CI*)
County-level variables			
Residence area (Ref. = Rural)			
Urban	2.23 (2.01–2.47)	1.50 (1.35–1.66)	1.44 (1.30–1.59)
Sociodemographic variables			
Sex (Ref. = Female)	1.73 (1.56–1.91)	1.11 (1.01–1.23)	1.05 (0.94–1.19)
Male			
Age group (years) (Ref. = 60–69 years)			
70–79	0.51 (0.45–0.58)	0.65 (0.59–0.73)	0.65 (0.58–0.73)
≥80	0.20 (0.15–0.26)	0.28 (0.22–0.37)	0.30 (0.23–0.40)
Educational level (Ref. = Illiteracy)			
Primary school		5.14 (4.020–6.57)	4.89 (3.85–6.23)
Junior high school and above		7.84 (6.15–10.00)	7.06 (5.56–8.97)
Annual household income (Ref. = Low)			
Medium		2.05 (1.78–2.34)	1.99 (1.72–2.29)
High		2.50 (2.13–2.93)	2.20 (1.88–2.57)
Marital status (Ref. = divorced/widowed/cohabitation)			
Married		1.11 (0.94–1.30)	1.10 (0.93–1.29)
Living alone (Ref. = No)			
Yes		0.78 (0.67–0.90)	0.77 (0.67–0.89)
Lifestyle variables			
Smoking (Ref. = No)			
Yes			1.02 (0.91–1.15)
Drinking (Ref. = No)			
Yes			1.14 (1.02–1.27)
Insufficient fruit and vegetable intake (Ref. = No)			
Yes			0.70 (0.64–0.77)
Excessive red meat intake (Ref. = No)			
Yes			1.04 (0.91–1.20)
Physical activity (Ref. = No)			
Yes			1.01 (1.01–1.01)

Model 1: Adjusted for sex, age, and residence area.

Model 2: Model 1 plus educational level, annual household income, marital status, and living alone.

Model 3: Model 2 plus smoking, drinking, insufficient fruit and vegetable intake, excessive red meat intake, and physical activity.

## Discussion

This study is the first to estimate the prevalence of healthy aging among older people in Henan Province. The results showed that only 6.1% of older adults met the criteria for healthy aging. This rate is lower than the national prevalence of 13.2% among Chinese adults aged ≥60 years, as reported by the China Health and Retirement Longitudinal Study (CHARLS) (15). It is also lower than the 15.8% prevalence among adults aged ≥ 65 years, as reported by the Healthy Aging Evaluation Longitudinal Study in China (HAELS) (13), a study that used the same definition of healthy aging as our study. The disparity is mainly attributed to the lower prevalence of high physical function in our study (39.0%), which is far less than the figures reported in the two comparative studies (70.2 and 79.3%, respectively). Zhou et al. (19) compiled data from 30 studies on healthy aging globally, reporting prevalence rates of 8.5% in Europe and 10.9% in the US. The discrepancy is also related to differences in high physical function, with prevalence rates of 57.3% in Europe and 49.0% in the US. As expected, our study found negative associations between healthy aging and both age and rural residence, which is consistent with previous studies (13, 19).

Subgroup analysis showed that the prevalence of healthy aging was higher in male individuals than in female individuals. This difference can be attributed to the higher prevalence of all five indicators, especially active social engagement (36.3% vs. 1.8%). These findings suggests that female individuals face more health problems, given their longer average life expectancy and higher prevalence of multimorbidity compared to male individuals (20). In addition, the effects of menopause and ovarian senescence contribute to decreased physical function, resulting in lower rates of healthy aging (21). Older adults with higher educational levels and better economic status had greater access to resources that promote healthy aging. This could be explained by their greater access to health knowledge, better awareness of health issues, more effective utilization of healthcare services, and the ability to access advanced medical resources to maintain a healthy lifestyle and prevent chronic diseases (22). Marital status and living alone also played significant roles in healthy aging. The higher prevalence of healthy aging among older individuals who were married or not living alone is mainly due to the support provided by their partners or other family members, including assistance with daily activities, emotional support, and opportunities for active social engagement (23), all of which are key factors contributing to healthy aging. Extensive evidence confirms that regular physical activity reduces the risk of multiple chronic diseases (24), which is crucial for promoting healthy aging. Similarly, insufficient consumption of fruits and vegetables is detrimental to healthy aging. This aligns with recommendations for chronic disease prevention, which emphasize regular physical exercise and an adequate intake of fresh fruits and vegetables (25). Therefore, older adults should be encouraged to engage in regular physical exercise, such as Tai Chi, Baduanjin, and swimming, to enhance physical function, alongside maintaining adequate daily intake of dietary fiber.

Unexpectedly, drinking was positively associated with healthy aging, consistent with a previous study (19), which contrasts with earlier conclusions that alcohol is a risk factor for multiple chronic diseases and increases all-cause mortality among older people (26, 27). In Chinese traditional culture, smoking and drinking often serve as social tools to enhance emotional communication and social support. However, these results are highly susceptible to reverse causality and survivor bias. Individuals in better health are more likely to engage in social activities and maintain a healthy diet, while those vulnerable to the adverse effects of these exposures may have been excluded due to death or severe disease. Furthermore, residual confounding by socioeconomic or broader lifestyle factors could not be fully controlled. Therefore, rigorous longitudinal studies are required to determine the causal relationship.

To the best of our knowledge, Henan Province is the first in China to conduct a cross-sectional study to evaluate the prevalence of healthy aging, providing evidence to support the adjustment of health management strategies. Second, participants were sampled according to the Seventh Population Census of Henan Province to ensure a representative population structure. However, several limitations should be considered when interpreting our findings. Casualty could not be confirmed due to the cross-sectional study design. Moreover, the exclusion of participants with significant hearing impairment may have introduced a potential source of selection bias. As hearing loss is a prevalent condition strongly associated with social isolation, cognitive decline, and lower health-related quality of life (28, 29), its exclusion likely resulted in a structurally healthier sample. Consequently, the prevalence of healthy aging was probably overestimated and may not be fully generalizable to the entire older population, which includes a substantial proportion with sensory impairments. Future studies should endeavor to employ inclusive assessment modalities to minimize this bias. In addition, the study was conducted in 2023, shortly after the end of the COVID-19 pandemic. This unprecedented event likely exerted a substantial, although complex, impact on the health and well-being of older adults in Henan Province. First, the pandemic and associated social restrictions may have had lasting effects on cognitive function, mental health outcomes (e.g., worsening cognitive function and increased prevalence of depression or anxiety, which could affect self-reported health measures) (30, 31) and health behaviors (e.g., reduced physical activity, unbalanced dietary patterns, increased alcohol consumption, and changes in sleep patterns) (32). Second, the high morbidity and mortality risks associated with COVID-19 among older people may have heightened population-wide awareness of vulnerability and preventive health, potentially influencing survey responses related to health perceptions and lifestyle behaviors (33). Third, the strain on healthcare systems during the pandemic could have affected the management of chronic conditions, indirectly impacting the health status of our study population. Although our cross-sectional design could not establish causality with respect to the pandemic, its potential impact remains an important consideration. Therefore, our results may reflect a unique post-pandemic baseline for healthy aging in this region. Future longitudinal studies are urgently needed to disentangle the acute and chronic effects of such a major public health crisis from the long-term trajectories of population aging.

## Conclusion

In conclusion, the true prevalence of healthy aging in Henan Province is likely lower than what we estimated in our study. The prevalence of healthy aging was higher among adults aged 60–69 years, male individuals, urban residents, married individuals, and individuals with higher educational and economic levels. These findings provided evidence to support the promotion of healthy aging, particularly among those living in rural areas. This requires a multi-dimensional approach involving healthcare, economic, and educational systems, with support from governmental and non-governmental organizations, communities, families, and individuals. Given the economic situation in Henan Province, it is advisable to incorporate the promotion of healthy aging into the National Demonstration Areas for Comprehensive Prevention and Control of Non-communicable Diseases and the China Healthy Lifestyle for All Initiative to maximize its public health impact.

## Data Availability

The raw data supporting the conclusions of this article will be made available by the authors, without undue reservation.
